# Development of Gene-Based SSR Markers in Winged Bean (*Psophocarpus tetragonolobus* (L.) DC.) for Diversity Assessment

**DOI:** 10.3390/genes8030100

**Published:** 2017-03-09

**Authors:** Quin Nee Wong, Alberto Stefano Tanzi, Wai Kuan Ho, Sunir Malla, Martin Blythe, Asha Karunaratne, Festo Massawe, Sean Mayes

**Affiliations:** 1 Biotechnology Research Centre, School of Biosciences, Faculty of Science, University of Nottingham Malaysia Campus, Jalan Broga, 43500 Semenyih, Selangor Darul Ehsan, Malaysia; khyx4asi@nottingham.edu.my (A.S.T.); waikuan@cffresearch.org (W.K.H.); festo.massawe@nottingham.edu.my (F.M.); 2 Crops For the Future, Jalan Broga, 43500 Semenyih, Selangor Darul Ehsan, Malaysia; asha.karunaratne@cffresearch.org; 3 Deep Seq, Faculty of Medicine and Health Sciences, Queen’s Medical Centre, University of Nottingham, Nottingham NG7 2UH, UK; sunir.malla@nottingham.ac.uk (S.M.); martinblythe@hotmail.com (M.B.); 4 Department of Export Agriculture, Faculty of Agricultural Sciences, Sabaragamuwa University of Sri Lanka, Belihuloya 70140, Sri Lanka; 5 School of Biosciences, Faculty of Science, University of Nottingham Sutton Bonington Campus, Sutton Bonington, Leicestershire LE12 5RD, UK

**Keywords:** *Psophocarpus tetragonolobus* (L.) DC., winged bean, SSR marker, transcriptome

## Abstract

Winged bean (*Psophocarpus tetragonolobus*) is an herbaceous multipurpose legume grown in hot and humid countries as a pulse, vegetable (leaves and pods), or root tuber crop depending on local consumption preferences. In addition to its different nutrient-rich edible parts which could contribute to food and nutritional security, it is an efficient nitrogen fixer as a component of sustainable agricultural systems. Generating genetic resources and improved lines would help to accelerate the breeding improvement of this crop, as the lack of improved cultivars adapted to specific environments has been one of the limitations preventing wider use. A transcriptomic de novo assembly was constructed from four tissues: leaf, root, pod, and reproductive tissues from Malaysian accessions, comprising of 198,554 contigs with a N50 of 1462 bp. Of these, 138,958 (70.0%) could be annotated. Among 9682 genic simple sequence repeat (SSR) motifs identified (excluding monomer repeats), trinucleotide-repeats were the most abundant (4855), followed by di-nucleotide (4500) repeats. A total of 18 SSR markers targeting di- and tri-nucleotide repeats have been validated as polymorphic markers based on an initial assessment of nine genotypes originated from five countries. A cluster analysis revealed provisional clusters among this limited, yet diverse selection of germplasm. The developed assembly and validated genic SSRs in this study provide a foundation for a better understanding of the plant breeding system for the genetic improvement of winged bean.

## 1. Introduction

Winged bean (*Psophocarpus tetragonolobus* (L.) DC.) (2*n* = 2*x* = 18) is a tropical perennial vine species, classified in the family of Fabaceae and subfamily of Papilionoideae, that is cultivated mainly at a subsistence scale in hot and humid countries across India, Southeast Asia, and the Western Pacific islands, with a presence in a number of African countries as well [1,2,3,4,5]. It is grown for its green pods, tuberous roots, and mature seeds, all of which have received attention for their nutritional content in the past, as comprehensively described in ‘*The Winged Bean—A high-protein crop for the tropics*’ from the National Academy of Science in 1981 [1]. Initial interest was drawn to high crude protein levels in seeds, which are comparable to soybean [6,7,8]. Its vining nature and nitrogen fixation activity have seen it used as a cover crop and also incorporated into rotation or intercropping systems [9,10,11]. As such, winged bean could be a good candidate for diversifying diets to improve nutritional security, based on complex and more sustainable agricultural systems [12]. Despite its potential, winged bean has received limited research investment for developing molecular tools that can support breeding programmes, until recently. Recent reports include the development of inter-Simple Sequence Repeats (iSSRs) and Randomly Amplified Polymorphic DNA (RAPD) markers for genetic diversity and for clonal fidelity analyses and two small transcriptomic assemblies derived from a mix of leaf, bud, and shoot of Sri Lankan accessions and leaf tissue from a Nigerian accession, respectively [13,14,15,16,17]. Given that winged bean is believed to be largely self-pollinated, heterozygosity would be expected to be low, although a formal assessment is needed and the species does produce large flowers, suggesting a contribution from insect pollination, as recorded by Erskine [18]. Thus, molecular breeding will facilitate utilisation of genetic resources in winged bean breeding, especially among accessions, through combining beneficial traits. Molecular markers that are tightly linked to important agronomic traits are a precondition for undertaking molecular breeding in plants. The genetic basis of traits in winged bean remains largely unexplored, and to date there has not been any genetic linkage map reported for this crop, although controlled crosses have been reported [19,20,21,22].

In this study, we generated RNA-seq data from four tissues (leaf, root, reproductive tissues, and pod) of six locally grown accessions, followed by the identification of SSR-containing sequences and validation of a subset of genic SSR markers. To our knowledge, this is the first application of within-species genic SSR markers in winged bean accessions. The data will help to begin the development of comprehensive genetic information and tools to facilitate future breeding programmes, as well as allow the levels of natural inbreeding to be determined, to allow appropriate breeding schemes to be devised. The transcriptome will allow us to gain a better understanding of the phylogenetic relationships between winged bean and other leguminous and model plants.

## 2. Material and Methods

### 2.1. Plant Material, RNA Extraction, Complementary DNA (cDNA) Library Construction, and Sequencing

A total of six locally grown winged bean accessions (two derived from Malaysian Agricultural Research and Development Institute (MARDI) and four from local planters) were grown from August to December 2012 at Lady Bird Farm, Broga, Semenyih, Malaysia (Latitude: 2.9394 N; Longitude: 101.8971 E; Altitude: 45m asl). RNA was extracted separately from leaf, root, pod, and reproductive tissue (comprising of bud and flower) by pooling the respective tissues from all the six accessions. Extraction was performed from different tissue groups separately using TRIzol Reagent (Thermo Fisher Scientific, Waltham, MA, USA) followed by another round of purification using RNeasy MinElute Cleanup Kit (Qiagen, Hilden, Germany) before library preparation.

Total RNA was measured using the Qubit RNA BR assay kit (Thermo Fisher Scientific, Waltham, MA, USA). A total of 5 µg of RNA was used for enrichment of mRNA using NEBNext Poly(A) mRNA Magnetic Isolation Module (New England Biolabs, Beverly, MA, USA). RNA fragmentation was done using NEBNext Magnesium RNA Fragmentation Module (New England Biolabs, Beverly, MA, USA). Illumina stranded whole transcriptome sequencing libraries were prepared through a dUTP approach using NEBNext Ultra Directional RNA Library Prep Kit for Illumina (New Engladn Biolabs, Beverly, MA, USA). Libraries were gel purified using 2% E-Gel SizeSelect (Thermo Fisher Scientific, Waltham, MA, USA) and quality control was performed using bioanalyser HS kit (Agilent biotechnologies, Palo Alto, CA, USA). Quantification was done using qPCR (quantitative polymerase chain reaction) (Kapa Biosystems, Woburn, MA, USA). Equimolar amounts of barcoded libraries were mixed and subjected to 250 bp paired-end run using MiSeq V2 chemistry (Illumina, San Diego, CA, USA) on Illumina MiSeq sequencing platform according to manufacturer’s instruction.

For simple sequence repeat (SSR) marker development, a total of nine plants from five accessions—each one representing a geographical origin—from the International Institute of Tropical Agriculture (IITA) genebank and MARDI, were used (as listed in Table 1 below). Two individuals were used per accession, which were collected after a cycle of single seed descent purification from January to June 2013 at the Lady Bird Farm, except for the Malaysian line.

### 2.2. De Novo Transcriptome Assembly and Microsatellite Identification

Adaptors and low quality reads (below Q20) were trimmed using Scythe and Sickle [23], respectively, using default settings. Trimmed reads from all tissues were pooled to assemble a combined de novo assembly with Trinity version 2.2.0 pipeline using the strand specificity option. Trinity assembled transcripts were annotated with Trinotate software suite version 1.1 [24], with a blast e-value threshold of 1 × 10^−5^ from NCBI-BLAST [25], HMMER/PFAM [26], SignalP [27], EMBL eggNOG [28], and Gene Ontology (GO) [29] databases. The data is deposited in NCBI Sequence Read Archive (BioProject ID PRJNA374598) under the accession number of SRP099538; SRR5252646 (root), SRR5252647 (reproductive tissue), SRR5252648 (pod), and SRR5252649 (leaf).

This was followed by the identification of microsatellites using MIcroSAtellite (MISA) Perl script program, based on a minimum number of repeats of six for di-, five for tri-, tetra-, penta- and hexa-nucleotide repeat motif (monomer repeats were excluded), whilst the maximum number of bases interrupting two SSRs in a compound microsatellite was 100 [30].

### 2.3. Microsatellite Markers Development and Scoring

Primer pairs were designed from sequences harbouring a minimum of 18-bases long microsatellites (i.e., minimum 9 and 6 repetitions for di- and tri-nucleotide motifs, respectively) (Table S1). Primer3 [31] and PrimerQuest (Integrated DNA Technologies, Coralville, Iowa, USA) were used for oligo design, with the latter used whenever the first was not able to design with standard parameters for the given sequence. Where possible, two pairs of primers were designed for a single target region, so as to have an alternative if the first pair failed to amplify the target region. DNA was extracted from the leaf of nine genotypes (Table 1) using a modified cetyltrimethylammonium bromide (CTAB) method [32]. In addition, an RNase digestion step was added and 1 volume of isopropanol was used instead of 2/3 volume. Primer screening and optimisation was carried out in a three primer system for fluorescent labelling [33] using an equimolar mixture of genotypes. Each 20 µL of PCR reaction consisted of 1× Buffer S, 200 µM dNTPs, 0.02 µM forward primer, 0.18 µM M13-dye labelled primer, 0.2 µM reverse primer, 20 ng of DNA, and 1 U of Taq DNA Polymerase (Vivantis, Subang Jaya, Selangor, Malaysia). The PCR was programmed for 3 min of initial denaturation at 94 °C, followed by 35 cycles of 1 min at 94 °C, 1 min at 60 °C, and 1 min at 72 °C, with a 10 min final elongation. Initial polymorphism evaluation of primers was performed across all genotypes using a long capillary fragment analyser (Advance Analytical Technologies – AATI, Ankeny, IA, USA) with default parameters, except for using 4 µL of samples, and 4 kV of separation voltage for 180 min per run. Data was then analysed with Advance Analytical PROSize 2.0 v1.3.1.1 (Advance Analytical Technologies – AATI, Ankeny, IA, USA) to identify potential SSR markers based on presence of polymorphic amplicons across genotypes.

PCR products of single genotypes from potentially polymorphic SSR markers were then separated using an ABI Genetic Analyser ABI3730XL using Peak Scanner v2 for scoring (Applied Biosystems, Foster City, CA, USA). Validated markers were subsequently characterised using Power Marker v3.5 [34] for major allelic frequency, alleles per marker, heterozygosity, and polymorphic information content (PIC).

### 2.4. Cluster Analysis

A hierarchical cluster analysis was performed with the Dice (also known as Nei and Li) similarity coefficient and unweighted pair-group method with arithmetic mean (UPGMA) algorithm in Genstat 18th Edition [35].

## 3. Results and Discussion

### 3.1. Transcriptome Assembly and In Silico Identification of Microsatellites

A total of four libraries generated 12.77 million reads of cleaned 250 bp paired ends (Table 2). The de novo assembly derived from all tissues produced a total of 198,554 contigs with an average size of 798 bp and an N50 of 1462 bp.

**Figure 1:**
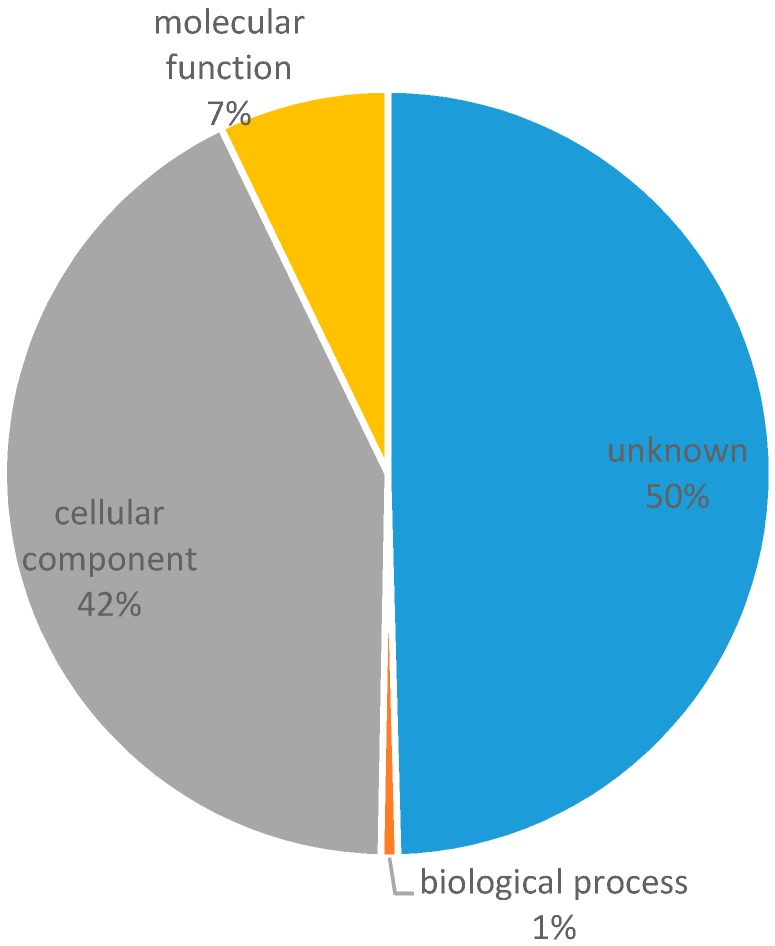
Distribution of first level gene ontology classification of the de novo assembly.

Out of 198,554 contigs, 138,958 (70.0%) could be annotated. Among them, 75,308 (54.2%), 69,172 (49.8%), 6499 (4.7%), 60,040 (43.2%), and 70,069 (50.4%) were found in NCBI-BLAST, HMMER/PFAM, SignalP, EMBL eggNOG, and GO databases, respectively, with no significant homology found from tmHMM on the prediction of transmembrane helices (Table S2). Figure 1 illustrates the abundance of transcripts classified based on gene ontology.

**Figure 2:**
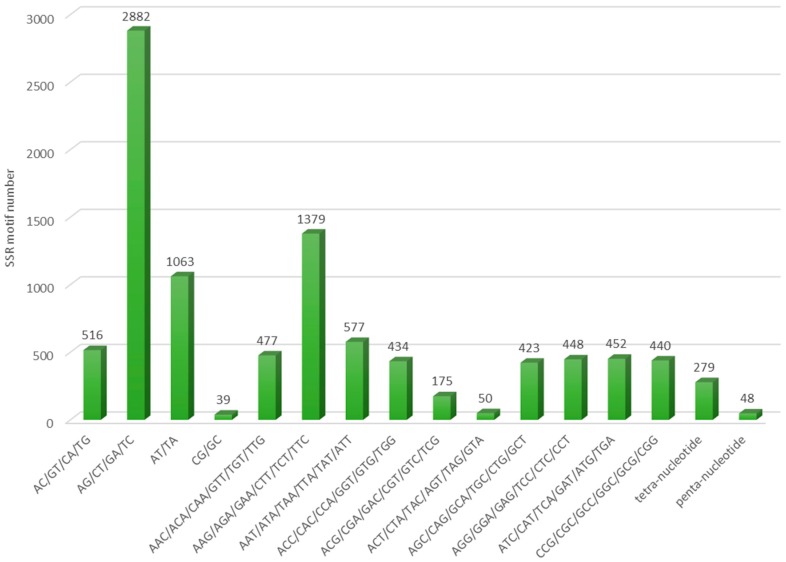
The number distribution of different microsatellite motif types identified. SSR, simple sequence repeat.

In this study, a total of 9682 putative SSR repeat motifs were identified from 8793 SSR containing sequences, which came from 4.4% of the total contig number in this assembly (Table S3). On average, there was one SSR locus for every 16.4 kbp of de novo assembly. After excluding mononucleotide motifs, trinucleotide repeats were the most abundant type (50.1%) (summarised in Table 3). This is consistent with Vatanparast et al.’s study [16], although hexamer motifs were not evaluated in this study. The most frequent dimer motifs were AG/GA/CT/TC type, followed by AT/TA, whereas for trimeric repeats, AAG/AGA/GAA/CTT/TCT/TTC were the most abundant (Figure 2 and Table 4). Both observations on the most common di- and tri-nucleotide repeat motif are in agreement with the winged bean transcriptome from Vatanparast et al. as well as with the soybean, medicago, and lotus Expressed Sequence Tag (EST)-SSR summarised by Jayashree et al. [16,36].

### 3.2 Development of SSR Markers and Cluster Analysis

A total of 56 (targeting 42 dimer-repeat regions) and 78 (targeting 53 trinucleotide SSR) primer pairs were designed. Subsequently, 20 dinucleotide SSR primers and 26 trinucleotide SSR primers gave good amplification products at the expected size. After polymorphism evaluation using all genotypes in this study, 18 validated SSR markers (8 for di-nucleotide and 10 for tri-nucleotide repeated motifs; Table S1) were scored and are summarised in Table 5. The low validation rate of polymorphic markers is likely to be partly due to the limited number of accessions screened, and should increase with more accessions covering a broader range of geographical origins. Residual heterozygosity could still be observed within each accession (shaded values in Table 5), even where a cycle of line purification in a controlled environment has been carried out, indicating that further cycles are needed to obtain homozygous lines, in particular for Tpt10, Tpt53, and M3. This data, along with the winged bean large flower size, also suggest that such a purification process may need to be carried out under an insect-proof enclosed environment. Using these markers, an average of 2.5 and 2.4 alleles per locus for di- and tri-nucleotide SSRs, respectively, was observed (Table 6). Individual PIC values varied from 0.16 to 0.67, which is comparable to recent legume studies in pigeonpea [37], mungbean [38], and common bean [39], although lower than in cowpea [40] and bambara groundnut [41].

**Figure 3:**
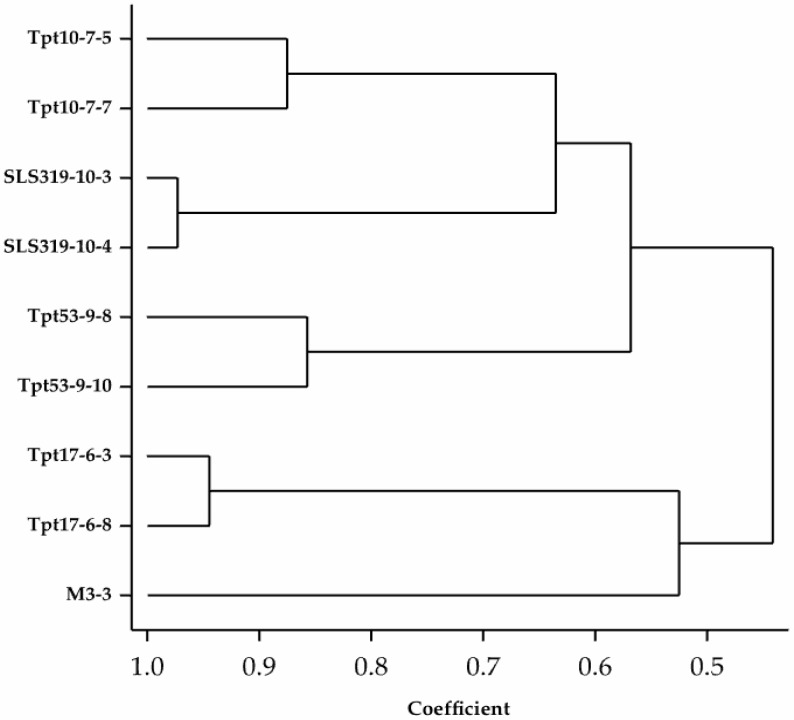
A dendrogram of the genetic relationship between genotypes from Papua New Guinea (Tpt10), Sri Lanka (SLS319), Bangladesh (Tpt53), Indonesia (Tpt17), and Malaysia (M3).

The cluster analysis from the SSR scores (Figure 3) showed a few clusters with the accessions originating from Papua New Guinea closely related to the Sri Lankan accession, but sharing the least similarity with the Malaysian and Indonesian materials, comparatively. To our knowledge, the genetic relationship between germplasm from Bangladesh and Malaysia are here investigated for the first time with molecular markers, and place the Bangladesh origin closer to the Sri Lankan and Papua New Guinean germplasm. Although the number of accessions used in this study is limited, they cover a reasonable range of germplasm from different origins.

## 4. Conclusions

A set of validated functional winged bean genic-SSR markers is reported here for the first time, to our best knowledge. The reported residual heterozygosity across screened genotypes has suggested that further investigation needs to be carried out on the rate of natural outcrossing in winged bean, in order to understand how genetic materials should be maintained, improved, and introduced into breeding programmes. The cluster analysis provides an initial insight into the potential for these markers to be used on a larger number of winged bean accessions, to carry out a more comprehensive diversity analysis with the evaluation of germplasm from genebanks and from commonly cultivated lines. Finally, this set of 18 microsatellite markers could also be used to contribute to genetic linkage maps in winged bean, with the integration of single nucleotide polymorphisms (SNPs) markers for higher density. Such a map would be the first backbone for linkage analysis and the genetic dissection of traits with agronomic importance in this legume.

## Figures and Tables

**Table 1:** Winged bean accessions (two individuals per origin, except Malaysian line) used and their origins.

Individuals	Origin
Tpt53-9-8 Tpt53-9-10	Bangladesh
Tpt17-6-3 Tpt17-6-8	Indonesia
M3-3	Malaysia
Tpt10-7-5 Tpt10-7-7	Papua New Guinea
SLS319-10-3 SLS319-10-4	Sri Lanka

**Table 2:** Summary statistics for the de novo assembled transcriptome.

Tissue	Leaf	Pod	Reproductive Tissue	Root
Number of raw read/base (bp)	3,150,356/1,544,004,822	3,973,092/1,868,456,680	3,544,968/1,719,303,632	3,873,893/1,859,527,511
Numbers of trimmed read/base (bp)	3,113,502/1,438,258,180	3,157,832/1,301,766,113	3,199,527/1,431,024,141	3,303,324/1,461,908,151
Number of contigs/base (bp)	198,554/158,382,439			
Average contig size (bp)	798			
N50	1462			

**Table 3:** In silico identification of microsatellites from the mixed tissue assembly. SSR, simple sequence repeat.

Total number of sequences examined	198,554
Total size of examined sequences (bp)	158,382,439
Total number of identified SSRs	9682
Number of SSR containing sequences	8793
Number of sequences containing more than one SSR	780
Number of SSRs present in compound formation	352
Number of dimer-repeat	4500
Number of trimer-repeat	4855
Number of tetramer-repeat	279
Number of pentamer-repeat	48

**Table 4:** Frequency distribution of di- and tri-nucleotide motif repeat in this de novo assembly.

	Number of Repeat Motif							Total	%
**Di-nucleotide**	5	6	7	8	9	10	>10		
AC/GT/CA/TG	-	256	138	63	37	12	10	516	11.5
AG/CT/GA/TC	-	995	543	330	391	434	189	2882	64.0
AT/TA	-	407	201	1167	113	107	68	1063	23.6
CG/GC	-	38	1	0	0	0	0	39	0.9
Total	-	1696 (37.7%)	883 (19.6%)	560 (12.4%)	541 (12.0%)	553 (12.3%)	267 (5.9%)	4500	
**Tri-nucleotide**									
AAC/ACA/CAA/GTT/TGT/TTG	279	131	50	17	0	0	0	477	9.8
AAG/AGA/GAA/CTT/TCT/TTC	612	405	351	11	0	0	0	1379	28.4
AAT/ATA/TAA/TTA/TAT/ATT	305	145	112	15	0	0	0	577	11.9
ACC/CAC/CCA/GGT/GTG/TGG	307	62	55	10	0	0	0	434	8.9
ACG/CGA/GAC/CGT/GTC/TCG	90	66	12	7	0	0	0	175	3.6
ACT/CTA/TAC/AGT/TAG/GTA	36	8	3	3	0	0	0	50	1.0
AGC/CAG/GCA/TGC/CTG/GCT	271	105	38	9	0	0	0	423	8.7
AGG/GGA/GAG/TCC/CTC/CCT	247	115	75	11	0	0	0	448	9.2
ATC/CAT/TCA/GAT/ATG/TGA	311	83	24	34	0	0	0	452	9.3
CCG/CGC/GCC/GGC/GCG/CGG	247	130	55	8	0	0	0	440	9.1
Total	2705 (55.7%)	1250 (25.7%)	775 (16.0%)	125 (2.6%)	0	0	0	4855	

**Table 5:** Scores of 18 SSR markers from nine winged bean individuals.

Marker	Papua New Guinea		Indonesia		Bangladesh		Sri Lanka		Malaysia
	Tpt10-7-5	Tpt10-7-7	Tpt17-6-3	Tpt17-6-8	Tpt53-9-8	Tpt53-9-10	SLS319-10-3	SLS319-10-4	M3-3
P27.2	205	199/205	199	205	205	205	205	205	205
P43.2	199	199	195	195	197	199	199	199	195
Pt1.1	335	335	339	339	339	335/339	335	335	339
Pt10	226/228	228	226	226	228	228	228	228	228
Pt14	358	358	352	352	350	350	358	358	354
Pt24	219	217/219	217	217	219	219	219	219	217
Pt7.2	426/432	426	426	426	428	428	426	426	426/428
WB17	198	198	198	198	198	194/198	198	198	198
Pt53	315	309	315	315	309/315	309/315	312	312	315
Pt58	255/261	255/261	261	261	261	261	261	261	261
Pt65.1	273	273	267	267	267	267	267	267	267/273
Pt67.1	293	293	296	296	293	293	296	296	293/296
Pt68.1	226	226	229	229	226	223/226	223	223/226	226/235
Pt76.1	203	203	203	203	209	209	209	209	209
Pt78.1	306/309	306/309	306	306	309	309	306	306	309
Pt85.1	276/279	276/279	276	276	276	276	276	276	279
Pt93.1	266	266	272	272	266/272	272	266	266	276
Pt99.2	189/195	189/195	195	195	189	189	189	189	195

**Table 6:** A summary of data analysis of 18 SSR markers. PIC, polymorphic information content.

Marker	SSR Motif	Major Allele Frequency	No. of Alleles	Heterozygosity	PIC
P27.2	TA	0.83	2	0.11	0.24
P43.2	TA	0.56	3	0	0.49
Pt1.1	CT	0.5	2	0.11	0.38
Pt10	TC	0.72	2	0.11	0.32
Pt14	TG	0.44	4	0	0.64
Pt24	GT	0.61	2	0.11	0.36
Pt7.2	TC	0.67	3	0.22	0.4
WB17	GA	0.94	2	0.11	0.1
Average dimer SSR markers		0.66	2.5	0.1	0.37
Pt53	CGC	0.56	3	0.22	0.53
Pt58	TAG	0.89	2	0.22	0.18
Pt65.1	CAG	0.72	2	0.11	0.32
Pt67.1	AGA	0.5	2	0.11	0.38
Pt68.1	AAC	0.5	4	0.33	0.59
Pt76.1	CGC	0.56	2	0	0.37
Pt78.1	AAC	0.56	2	0.22	0.37
Pt85.1	GCG	0.78	2	0.22	0.29
Pt93.1	TGT	0.5	3	0.11	0.5
Pt99.2	TTC	0.56	2	0.22	0.37
Average trimer SSR marker		0.61	2.4	0.18	0.39

## Supplementary Materials

The following are available online at www.mdpi.com/2073-4425/8/3/100/s1, Table S1: the sequences of forward and reverse SSR-primers used in this study; Table S2: Functional annotation of assembled transcripts; Table S3: Identified microsatellites.
