# Supplementary material for: Development of Gene-Based SSR Markers in Winged Bean (Psophocarpus tetragonolobus (L.) DC.) for Diversity Assessment
Source: Genes (Basel). 2017 Mar 9;8(3):100. doi: 10.3390/genes8030100 (PMC5368704; doi:10.3390/genes8030100)
Supplement: Supplementary file 1 [file genes-08-00100-s001.zip › Supplementary files/Table S1.docx]

**Supplementary Files:**

**Table S1.** The sequences of forward and reverse SSR-primers used in this study.

| **SSR Motif** | **Marker** | **Primer sequence 5’ -> 3’** |
| --- | --- | --- |
|  |  |  |
| TA | P27.2F | CCTCCCAGATCAACCACTACTTTC |
|  | P27.2R | GGATAGGCATGGGTTCCTTTACTC |
| TA | P43.2F | GGAGTGTAACTAAGTATCGTGTATGG |
|  | P43.2R | AGATGCTCTTGGTTTACATCATTTG |
| CT | Pt1.1F | GCACCTAACAACCAATCTAAC |
|  | Pt1.1R | GATACAATCACCGTGTTGCTTCAA |
| TC | Pt10F | CCATTCACCACCGTATTCACTATTC |
|  | Pt10R | CACAGAATCAGAAATCACGACAGG |
| TG | Pt14F | TAAACTCGGTAAGTGTGTTCTTGAG |
|  | Pt14R | GAGACGCAAAGAGTAAGCAATAAG |
| GT | Pt24F | GAAAGAAAGAGGTTTGTTTGGAAGG |
|  | Pt24R | CCTTCTATTTCTCTCAACCAGTCTTC |
| TC | Pt7.2F | CCCTCCTTCTTGTTTAGTAGTGTAA |
|  | Pt7.2R | CCTGCAGTTCTGTTCTGTTTG |
| GA | WB17F | CATAATGGGTTTCATGGCAATGTG |
|  | WB17R | GCAAAGTTTGTGAATAGCTCCATAAC |
| CGC | Pt53F | TTCAATTCCGACTTCTTAATCCATTCCCCGAA |
|  | Pt53R | CTTCGTAGGTTTGATTTGAAGACGCCGAAG |
| TAG | Pt58F | TCTGAGGAGCTGAAAAGTTTGGCGCTTC |
|  | Pt58R | TGAACTTGCATCATGGGTTGGATCAGTC |
| CAG | Pt65.1F | CCTTTCCTGCAACACAAACCAAAC |
|  | Pt65.1R | GAGGTCCTTAATTTCCTCGAACAAGTG |
| AGA | Pt67.1F | CAGCCCGTGAGTAAGTTGCATTAG |
|  | Pt67.1R | GCTCTTCTTCAACCTCTTCTTCTTCTTC |
| AAC | Pt68.1F | CTCCGCGAGAACCTTAATCAATCG |
|  | Pt68.1R | GCACAGCATAGCTTCTACTTCTGG |
| CGC | Pt76.1F | CACTCCAATTTCAACCATGCCCTTT |
|  | Pt76.1R | GAAGAACACGACCACCAGGATGAC |
| AAC | Pt78.1F | TAGCAATGAAGAAGTGGAGTACGGAATAG |
|  | Pt78.1R | GCAATTTGGGAATGTGGTGGGTAA |
| GCG | Pt85.1F | CCTGAATGAGAAGCTGAAACGAAACG |
|  | Pt85.1R | GAGGTGCCATGCGATACATATACTCC |
| TGT | Pt93.1F | CACCACACCATTCTCTTCCTCTTC |
|  | Pt93.1R | CCCATTTCACTGATTCAAATCATCACC |
| TTC | Pt99.2F | TCATCTTTCCATGGCTTCCACTCC |
|  | Pt99.2R | CAAGTGATTATGATGAGGGCTTTGTGC |
